# Binge-Eating-Störung – State of the Art

**DOI:** 10.1007/s00115-025-01818-6

**Published:** 2025-03-17

**Authors:** Katrin Giel, Stephan Zipfel, Kathrin Schag

**Affiliations:** 1 Abteilung für Psychosomatische Medizin & Psychotherapie, Medizinische Universitätsklinik Tübingen, Osianderstr. 5, 72076 Tübingen, Deutschland; 2 Kompetenzzentrum für Essstörungen Tübingen (KOMET), Tübingen, Deutschland; 3 Deutsches Zentrum für Psychische Gesundheit (DZPG), Standort Tübingen, Deutschland

**Keywords:** Adipositas, Essanfälle, Essstörung, Kontrollverlust, Psychotherapie, Obesity, Binge eating episodes, Eating disorder, Loss of control, Psychotherapy

## Abstract

**Hintergrund:**

Essstörungen sind komplexe psychische Störungen, deren Prävalenz insbesondere seit der Corona-Pandemie weiter zunimmt. Die Binge-Eating-Störung wurde als Diagnose neu in das Diagnostic and Statistical Manual of Mental Disorders 5 (DSM-5) und die International Statistical Classification of Diseases and Related Health Problems 11 (ICD-11) aufgenommen.

**Ziel der Arbeit:**

Wir geben einen State-of-the-Art-Überblick zu diagnostischen Kriterien, Psychopathologie, Differenzialdiagnostik, Epidemiologie, Komorbiditäten, Entstehungs- und Aufrechterhaltungsfaktoren, Therapie und Versorgungssituation der Binge-Eating-Störung.

**Material und Methoden:**

Es wurde ein narratives Review erarbeitet.

**Ergebnisse:**

Die Binge-Eating-Störung ist durch wiederkehrende Essanfälle mit Kontrollverlust gekennzeichnet. Sie ist die häufigste Essstörung in der Allgemeinbevölkerung und häufig mit Übergewicht oder Adipositas vergesellschaftet. Neurobiologische Modelle sehen Veränderungen im Bereich der Emotionsregulation, Belohnungsverarbeitung und Impulskontrolle als ätiologische Beiträge zur Binge-Eating-Störung. Psychotherapie ist der Behandlungsansatz der ersten Wahl bei der Binge-Eating-Störung, die häufig unentdeckt und unversorgt bleibt. Ein Grund hierfür sind erlebte oder befürchtete Stigmatisierung, Scham und Schuldgefühle der Betroffenen.

**Diskussion:**

Da die Diagnose einer Binge-Eating-Störung die Therapiewahl und Prognose sowohl der Essstörung als auch einer potenziell komorbiden Adipositas beeinflusst, ist das aktive Abklären des Essverhaltens und einer möglichen Essstörung im Rahmen einer motivierenden Gesprächsführung essenziell.

## Abstract (en)

**Background:**

Eating disorders are complex mental disorders the prevalence of which has continued to increase, particularly since the coronavirus pandemic. Binge Eating Disorder has been newly included as a diagnosis in the Diagnostic and Statistical Manual of Mental disorders 5 (DSM-5) and the International Classification of Diseases 11th revision (ICD-11).

**Aim of the work:**

We present a state of the art overview of diagnostic criteria, psychopathology, differential diagnostics, epidemiology, comorbidities, developmental and maintenance factors, treatment and care situation of binge eating disorder.

**Material and methods:**

A narrative review is provided.

**Results:**

Binge eating disorder is characterized by recurrent binge eating with loss of control. It is the most common eating disorder in the general population and is often associated with overweight or obesity. Neurobiological models see changes in the areas of emotion regulation, reward processing and impulse control as etiological contributions to binge eating disorder. Psychotherapy is the treatment of choice for binge eating disorder, which often goes undetected and untreated. A reason for this is the experienced or feared stigmatization, shame and feelings of guilt of those affected.

**Discussion:**

As the diagnosis of a binge eating disorder influences the choice of treatment and the prognosis of both the eating disorder and a potentially comorbid obesity, active clarification of the eating behavior and a possible eating disorder in the context of motivational interviewing is essential.

Die Binge-Eating-Störung (BES) wurde 2013 erstmalig in das Diagnostische und statistische Manual psychischer Störungen (DSM-5) aufgenommen und findet sich mittlerweile auch als neue Diagnose in der International Statistical Classification of Diseases and Related Health Problems 11 (ICD-11). Die BES ist durch wiederkehrende Essanfälle mit Kontrollverlust gekennzeichnet, wobei keine regelmäßigen gegensteuernden Maßnahmen nach den Essanfällen ergriffen werden. Sie ist die häufigste Essstörung in der Allgemeinbevölkerung.

## Diagnostische Kriterien und Psychopathologie

Einen Überblick über die aktuellen Diagnosekriterien der BES gibt Tab. 1. ICD-11- und DSM-5-Klassifikation stimmen in den Kernaussagen grundsätzlich überein. Die ICD-11-Kriterien sind jedoch weitaus breiter formuliert bezüglich der Häufigkeit der Essanfälle und sehen den subjektiven Kontrollverlust im Vordergrund der Psychopathologie, wohingegen die Nahrungsmenge im Rahmen eines Essanfalls im Vergleich zur DSM-5-Auffassung in den Hintergrund tritt [9]. Die DSM-5-Klassifikation spezifiziert weitere Charakteristika von Essanfällen, die auch wichtig sind, um vermehrtes Essen im Rahmen anderer gesellschaftlicher Bezüge (etwa bei Festlichkeiten) von pathologischem Essverhalten abzugrenzen. Beispielsweise essen viele Betroffene im Rahmen eines Essanfalls alleine aus Scham über ihr Essverhalten.

**Tab. 1:** Diagnosekriterien der Binge-eating-Störung nach dem Diagnostic and Statistical Manual of Mental Disorders 5 (DSM‑5; [1]) und der International Statistical Classification of Diseases and Related Health Problems 11 (ICD-11 [30])

DSM‑5	ICD-11
*A: Wiederholte Episoden von Essanfällen*	Die Binge-eating-Störung ist durch häufige, wiederkehrende Episoden von Essanfällen gekennzeichnet (z. B. einmal pro Woche oder öfter über einen Zeitraum von mehreren Monaten)
Ein Essanfall ist gekennzeichnet durch	Ein Essanfall ist ein bestimmter Zeitraum, in dem die betroffene Person subjektiv die Kontrolle über das Essen verliert, deutlich mehr oder anders isst als gewöhnlich und sich nicht in der Lage fühlt, mit dem Essen aufzuhören oder die Art oder Menge der verzehrten Lebensmittel zu begrenzen
(1) Verzehr einer erheblich größeren Nahrungsmenge als die meisten Menschen innerhalb eines vergleichbaren Zeitraumes unter vergleichbaren Bedingungen essen würden und	
(2) das Gefühl, während der Episode die Kontrolle über das Essverhalten zu verlieren	
*B: Die Essanfälle treten gemeinsam mit mindestens drei der folgenden Symptome auf:*	„Binge eating“ wird als sehr belastend empfunden und oft von negativen Gefühlen wie Schuld oder Ekel begleitet
(1) Wesentlich schneller essen als normal	
(2) Essen bis zu einem unangenehmen Völlegefühl	
(3) Essen großer Nahrungsmengen, wenn man sich körperlich nicht hungrig fühlt	
(4) Alleine essen aufgrund von Scham über die Menge, die man isst	
(5) Ekelgefühle gegenüber sich selbst, Deprimiertheit oder große Schuldgefühle nach dem übermäßigen Essen	
*C: Es besteht ein deutlicher Leidensdruck wegen der Essanfälle*	Es besteht ein ausgeprägter Leidensdruck aufgrund der Essanfälle oder eine erhebliche Beeinträchtigung in persönlichen, familiären, sozialen, schulischen, beruflichen oder anderen wichtigen Bereichen
*D: Die Essanfälle treten im Durchschnitt mindestens einmal pro Woche über einen Zeitraum von 3 Monaten auf*	–
*E: Die Essanfälle treten nicht gemeinsam mit wiederholten unangemessenen kompensatorischen Maßnahmen (Differenzialdiagnose Bulimia nervosa) und nicht ausschließlich im Verlauf einer Bulimia nervosa oder Anorexia Nervosa auf*	Anders als bei Bulimia nervosa folgen auf die Essanfälle keine regelmäßig unangemessenen kompensatorischen Verhaltensweisen, die eine Gewichtszunahme verhindern sollen (z. B. selbst herbeigeführtes Erbrechen, Missbrauch von Abführmitteln oder Einläufen, exzessives Sporttreiben)

## Differenzialdiagnostik

Im Zusammenhang mit den relativ breit formulierten Diagnosekriterien der BES in der ICD-11, die den subjektiven Kontrollverlust in den Vordergrund stellen, wurde auf die schwierige diagnostische Abgrenzung eines Essanfalls von anderen maladaptiven Essmustern hingewiesen. Essmuster wie z. B. das sogenannte „grazing“, bei dem kleine Nahrungsmengen über einen längeren Zeitraum aufgenommen werden, treten insbesondere bei Menschen mit Übergewicht und Adipositas häufig auf, sind aber nicht mit einer Essstörung gleichzusetzen [9]. Da es keine Standards zur Erfassung von erlebtem Kontrollverlust gibt, kann es zu Über- oder auch Unterdiagnostik kommen.

Die BES ist im Spektrum der psychischen Störungen insbesondere von der Essstörung Bulimia nervosa zu unterscheiden (Tab. 1). Essanfälle mit Kontrollverlust treten bei beiden Essstörungen auf, allerdings werden bei der BES im Gegensatz zur Bulimia nervosa keine regelmäßigen kompensatorischen Maßnahmen im Anschluss an die Essanfälle ergriffen, wie beispielsweise selbstinduziertes Erbrechen oder exzessives Sporttreiben.

Insbesondere im Rahmen von affektiven Störungen, Substanzgebrauchsstörungen und der Borderline-Persönlichkeitsstörung kann es ebenfalls zu vermehrter Nahrungsaufnahme und impulsivem Essen kommen, die von einer primären BES abgegrenzt werden müssen [9].

Differenzialdiagnostisch sind außerdem neurologische, endokrine und genetische Erkrankungen in Betracht zu ziehen, die für impulsives Essverhalten verantwortlich sein können [9].

## Epidemiologie

Aktuelle bevölkerungsbasierte epidemiologische Daten berichten eine Lebenszeitprävalenz der BES von bis zu 6,1 % bei Jugendlichen und von bis zu 1,8 % für erwachsene Frauen sowie bis zu 0,7 % für erwachsene Männer [18]. Die Prävalenz von BES, Essanfällen oder subklinischen Formen von BES ist deutlich höher in klinischen Stichproben, die Therapieangebote zur Gewichtsreduktion aufsuchen, inklusive adipositaschirurgischer Maßnahmen [15, 21].

## Komorbiditäten

Sowohl körperliche als auch psychische Komorbiditäten sind bei der BES häufig. So erfüllten in einer bevölkerungsrepräsentativen Studie in den USA 94 % der Betroffenen mit BES mindestens noch eine weitere psychische Diagnose, am häufigsten die einer affektiven Störung, gefolgt von Traumafolgestörungen und anderen Angststörungen [29]. Impulskontrollstörungen werden ebenfalls häufig bei Menschen mit BES diagnostiziert, darunter beispielsweise substanzassoziierte Erkrankungen oder Verhaltenssüchte, aber auch die Bordeline-Persönlichkeitsstörung [9], was auf eine mögliche gemeinsame Disposition dieser Störungen hinweist.

Die BES ist sehr häufig mit Übergewicht, Adipositas und dem metabolischen Syndrom vergesellschaftet [4, 29]. In diesem Zusammenhang treten als häufige somatische Komorbiditäten Bluthochdruck und Diabetes mellitus auf [29]. Menschen mit BES berichten darüber hinaus häufig gastrointestinale Beschwerden, muskuloskelettale Symptome, und sie haben ein höheres Risiko für eine Reihe von Tumorerkrankungen [9], wobei unklar bleibt, welche Rolle spezifisch Essanfälle für das erhöhte Tumorrisiko spielen in Abgrenzung zur Adipositas.

## Entstehungs- und Aufrechterhaltungsfaktoren

Ein umfassendes Ätiologiemodell der BES fehlt bislang. Forschung zu Entstehungs- und Aufrechterhaltungsfaktoren der BES ist aktuell vor allem von neurobiologischen Ansätzen geprägt [19].

Wie bei anderen psychischen Störungen auch, besteht eine genetische Disposition für BES, die unter anderem in einer familiären Häufung deutlich wird [17]. Genetische Befunde weisen darauf hin, dass BES mit einer Reihe anthropometrischer Marker assoziiert ist, die auch für Adipositas eine Rolle spielen und für die Gewichtsregulation wichtig sind [16]. Eine aktuelle genomweite Assoziationsstudie berichtet darüber hinaus BMI(Body-Mass-Index)-unabhängige genetische Überlappungen zwischen BES einerseits sowie Depression, Neurotizismus und Impulsivität [3].

Neurobiologische Evidenz legt nahe, dass für die Entstehung und Aufrechterhaltung der BES Dysregulationen in den Bereichen Emotionsregulation, Belohnungsverarbeitung und Impulskontrolle mit verantwortlich sind [9, 19]. Damit bestehen auch Übereinstimmungen zu neuesten genetischen Befunden zur Rolle von Impulsivität bei der BES [3], da die komplexe Interaktion von Belohnungsverarbeitung, Emotionsregulation und Impulskontrolle als Kern der Persönlichkeitseigenschaft Impulsivität angesehen wird.

**Abb. 1:**
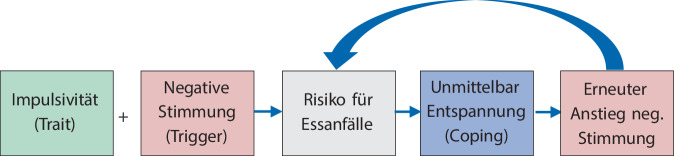
Prozessmodell bei Essanfällen

Von BES Betroffene berichten generell mehr negative Emotionen und nutzen eher dysfunktionale Emotionsregulationsstrategien [20]. Auf der Mikroebene gehen häufig negative Emotionen einem Essanfall unmittelbar voraus, sodass sich innerhalb eines individuellen Störungsmodells häufig Essanfälle als eine dysfunktionale kurzfristige Strategie zur Bewältigung dieser Gefühle identifizieren lassen [22], die aber langfristig zu erheblichen negativen Konsequenzen führt oder mittelfristig erneut negative Emotionen in Form von Scham- und Schuldgefühlen auslöst ([25]; Abb. 1).

Menschen mit BES reagieren sensitiver auf Nahrungsreize und empfinden diese als stark belohnend und suchen sie dementsprechend auch verstärkt auf [10, 27]. Sie haben größere Schwierigkeiten, ihr Verhalten zu hemmen, insbesondere, wenn sie mit diesen belohnenden Reizen konfrontiert sind [10, 27]. Das Zusammenspiel dieser beiden Komponenten lässt sich auch als erhöhte nahrungsbezogene Impulsivität beschreiben, in der sich Menschen mit BES von Menschen mit Adipositas ohne BES unterscheiden [4, 10, 27].

All diese Befunde spiegeln sich auch auf neurobiologischer Ebene wider. Funktionelle Bildgebungsstudien belegen, dass Betroffene mit BES eine veränderte Aktivierung verschiedener Hirnareale zeigen, und zwar insbesondere in Bereichen, die für die Steuerung des Energiehaushaltes zuständig sind (Hypothalamus), für die Belohnungsverarbeitung (u. a. Amygdala, ventrales Striatum, orbitofrontaler Kortex) und für die Hemmkontrolle (u. a. präfrontaler Kortex; [4, 9, 19]). Negative Stimmung wiederum kann präfrontale Bereiche inhibieren, wodurch subkortikale Aktivierungen wie beispielsweise im mesolimbischen System eher in Verhalten umgesetzt werden können [11]. Es ist darüber hinaus davon auszugehen, dass auch Veränderungen des Mikrobioms und in der Darm-Hirn-Kommunikation einen Beitrag zu BES leisten, beispielsweise über dysregulierte Peptidhormonsekretion, die wiederum die Hunger-Sättigungs-Regulation und die zentralnervös homöostatische Steuerung verändert [4, 9]. Hierzu liegen aber noch kaum Befunde vor.

**Abb. 2:**
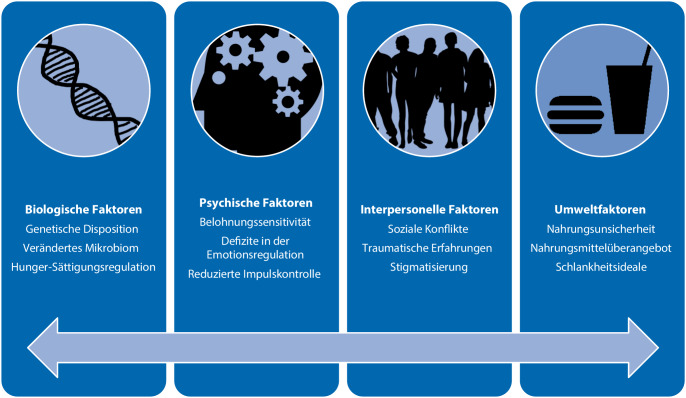
Auswahl an biologischen, psychischen, sozialen und umweltbezogenen Faktoren, die aktuell in der Entstehung und Aufrechterhaltung einer Binge-Eating-Störung diskutiert werden

Die nosologische Diskussion um die Konzeptualisierung der noch recht neuen Diagnose BES hält an [9]. Ein komplexes biopsychosoziales Entstehungsmodell psychischer Erkrankungen geht davon aus, dass die oben dargestellten (neuro)biologischen ätiologischen Faktoren ergänzt werden um weitere psychische und soziale Entstehungs- und Aufrechterhaltungsfaktoren (Abb. 2). Die Forschung zu Risikofaktoren der BES gibt hier erste Hinweise, beispielsweise zur Rolle von Missbrauchs- und Gewalterfahrungen, Armut und Nahrungsunsicherheit, aber auch einem Nahrungsmittelüberangebot und einem überhöhten Schlankheitsideal in der Entstehung von BES [9].

## Therapie

Da die BES sehr häufig mit erhöhtem Körpergewicht und Adipositas einhergeht, ist zunächst eine Abwägung und Festlegung zentraler Therapieziele wesentlich. Einige Menschen mit BES suchen primär Hilfe auf, weil sie Gewicht reduzieren möchten. Gleichzeitig empfehlen aktuelle Behandlungsleitlinien eine Normalisierung des Essverhaltens vor einer gewichtsorientierten Therapie zu priorisieren [12, 24]. Rationale hierfür ist unter anderem die Sorge, dass eine restriktive Ernährung im Rahmen konservativer Gewichtsreduktion Essanfälle eher begünstigen kann. Metaanalytisch zeigt sich, dass PatientInnen mit BES zwar grundsätzlich von evidenzbasierten Gewichtsreduktionsprogrammen profitieren, jedoch nicht hinsichtlich der Reduktion der Essanfälle [13]. Im Mittel nehmen PatientInnen mit BES in Gewichtsreduktionsprogrammen auch weniger Gewicht ab als Teilnehmende ohne BES und haben ein signifikant höheres Risiko, das Programm abzubrechen [8].

Internationale Behandlungsleitlinien empfehlen Psychotherapie als Behandlungsansatz der ersten Wahl bei BES [12, 24], in der Regel im ambulanten Setting. Die weitreichendste Evidenz für die Wirksamkeit bei BES besteht derzeit für die kognitive Verhaltenstherapie [13, 23], aber auch für die interpersonelle Psychotherapie und die dialektisch-behaviorale Psychotherapie liegen mittlerweile belastbare Wirksamkeitsnachweise bei der BES vor [9]. Laut einer aktuellen Metaanalyse erreicht rund die Hälfte der PatientInnen Abstinenz von Essanfällen innerhalb von 12 Monaten nach Beginn einer psychotherapeutischen Behandlung [14].

Im Bereich der Pharmakotherapie ist in Europa derzeit kein Medikament für die BES zugelassen, während das Stimulans Lisdexamfetamin in den USA für BES die Zulassung erhalten hat [9, 23]. Es ist eine Reihe verschiedener Wirkstoffe bei BES untersucht worden [9], wobei metaanalytisch die kurzzeitige Wirksamkeit von Antidepressiva und Lisdexamfetamin sowohl hinsichtlich eines verbesserten Essverhaltens als auch einer verbesserten psychischen Komorbidität gezeigt wurde [23]. Allerdings fehlen mittel- und langfristige Daten zur Wirksamkeit von Medikamenten bei BES sowie direkte Vergleiche zu Psychotherapie, es wurden Nebenwirkungen und niedrigere Akzeptanz berichtet [9, 14, 23].

Im Zuge des Einsatzes neuer Stoffklassen wie GLP-1(„glucagon-like peptide-1“)-Analoga für die erfolgreiche Gewichtsreduktion könnte es auch zu neuen Entwicklungen bei der Therapie der BES kommen. Erste Studienergebnisse deuten darauf hin, dass GLP-1-Analoga sich positiv auswirken können auf die Häufigkeit von Essanfällen, dem Verlangen nach Essen und auf psychische Komorbiditäten [2]. Randomisiert-kontrollierte Studien zur Wirksamkeit bei BES stehen aber noch aus.

Im Einklang mit aktuellen Befunden zur Neurobiologie der BES wird darüber hinaus an innovativen Therapieansätzen gearbeitet, hauptsächlich im Bereich der Psychotherapie [26], neurokognitiver Trainingsansätze und der Neuromodulation [6].

Digitale Ansätze und Dissemination können den Zugang zu evidenzbasierten Therapien erleichtern. Insbesondere im Bereich der angeleiteten Selbsthilfe wurden digitale Programme für die Behandlung der BES entwickelt [24]. Darüber hinaus spielen digitale Anwendungen auch in der Diagnostik eine Rolle, etwa zur Erfassung des Essverhaltens, der Essstörungssymptomatik oder eines anderen Gesundheitsverhaltens. Für viele Betroffene spielen mittlerweile Inhalte in sozialen Medien eine Rolle für die Aufrechterhaltung oder Veränderung ihrer Essstörung [24].

## Versorgungssituation

Daten aus verschiedenen Gesundheitssystemen zeigen, dass die BES oft unentdeckt bleibt und Menschen mit BES unversorgt sind [18] – dies hat vielschichtige Gründe. Ein Aspekt ist dabei die Vergesellschaftung der BES mit Übergewicht und Adipositas. Viele Personen mit BES suchen primär ein Gewichtsreduktionsprogramm auf [21], oft ohne sich darüber bewusst zu sein, dass sie eine Essstörung haben. Nur ein Bruchteil sucht in Bezug auf die BES Hilfe [5], und nur ein Teil erhält die richtige Diagnose und Therapie [28]. Menschen mit Essstörungen berichten, die Haupthürde, sich ins Gesundheitssystem zu begeben, bestehe für sie in erlebter oder befürchteter Stigmatisierung, Scham und Schuldgefühlen [7].

## Fazit für die Praxis

- Die Binge-Eating-Störung (BES) ist die häufigste Essstörung in der Allgemeinbevölkerung.
- Sie geht häufig mit Übergewicht und Adipositas einher.
- Die Binge-Eating-Störung bleibt häufig unentdeckt, Betroffene sind unterversorgt und stigmatisiert. Daher ist ein aktives Erfragen des Essverhaltens und Abklären einer möglichen Essstörung besonders wichtig.
- In der Gesprächsführung sollte eine wertfreie und motivierende Haltung eingenommen werden.
- Hinweise auf erlebten Kontrollverlust beim Essen sind Gefühle, nicht kontrollieren zu können, was oder wieviel man ist, das Essen nicht unterbrechen oder stoppen zu können, ein intensives Verlangen nach Essen („craving“) sowie Automatismen bei Essanfällen, denen die betroffene Person nichts aktiv entgegensetzt.
- Eine BES-Diagnose beeinflusst die Therapiewahl und Prognose sowohl der Essstörung als auch einer potenziell komorbiden Adipositas.
- Psychotherapie ist der Behandlungsansatz der ersten Wahl bei BES.
- Therapieziele bezüglich des Essverhaltens und einer möglichen Gewichtsreduktion sollten frühzeitig gemeinsam festgelegt werden.
